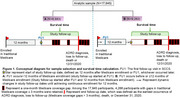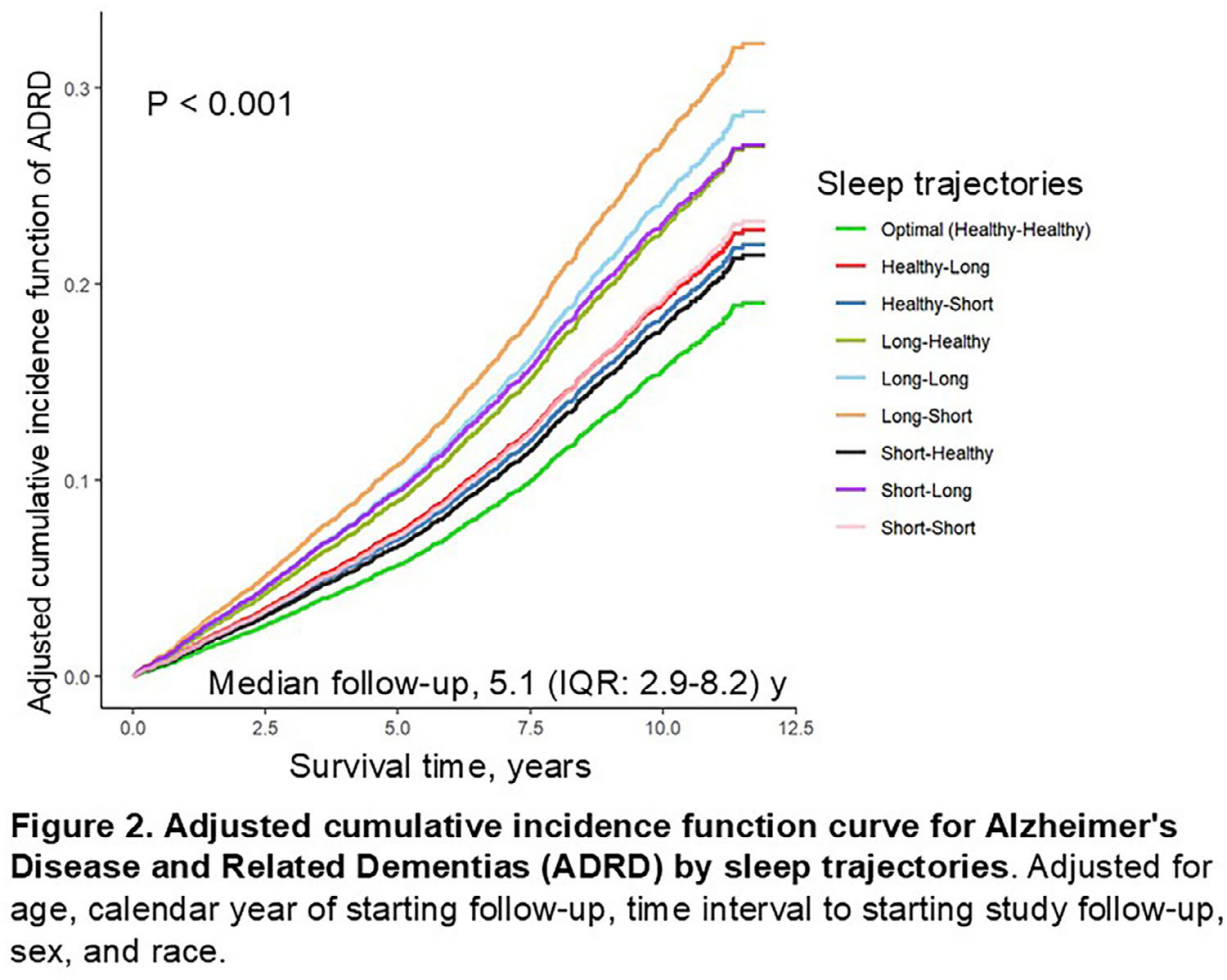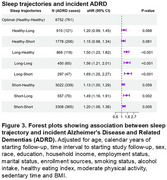# Sleep duration trajectories and Alzheimer's Disease and Related Dementias risk: The Southern Community Cohort Study

**DOI:** 10.1002/alz70860_105914

**Published:** 2025-12-23

**Authors:** Hui Shi, Loren Lipworth, Qian Xiao, Xijing Han, Michael Mumma, Laura Keohane, Danxia Yu, Corey J Bolton, Derek B. Archer, Timothy J. Hohman, Kelsie M Full

**Affiliations:** ^1^ Vanderbilt Memory and Alzheimer's Center, Nashville, TN, USA; ^2^ Vanderbilt University Medical Center, Nashville, TN, USA; ^3^ The University of Texas Health Science Center at Houston, Houston, TX, USA; ^4^ Vanderbilt Memory and Alzheimer's Center, Nashville, TN, USA; ^5^ Vanderbilt Genetics Institute, Vanderbilt University Medical Center, Nashville, TN, USA; ^6^ Vanderbilt Memory & Alzheimer's Center, Vanderbilt University Medical Center, Nashville, TN, USA; ^7^ Department of Neurology, Vanderbilt Memory & Alzheimer's Center, Vanderbilt University Medical Center, Nashville, TN, USA; ^8^ Vanderbilt Brain Institute, Vanderbilt University Medical Center, Nashville, TN, USA; ^9^ Vanderbilt Memory and Alzheimer's Center, Vanderbilt University School of Medicine, Nashville, TN, USA; ^10^ Vanderbilt Memory and Alzheimer's Center, Vanderbilt University Medical Center, Nashville, TN, USA; ^11^ Department of Medicine, Vanderbilt University Medical Center, Nashville, TN, USA

## Abstract

**Background:**

Both short and long sleep durations are adversely associated with Alzheimer's Disease and Related Dementias (ADRD). However, few studies have examined trajectories of sleep duration over time and whether they are associated with ADRD risk. This study aimed to examine long‐term sleep duration trajectories and risk of ADRD in a large community‐based cohort of predominately low‐income Black and White adults.

**Method:**

Among participants from the Southern Community Cohort Study (SCCS) who were 65 years of age or older and enrolled in Medicare, we identified incident ADRD cases using Medicare claims data. Sleep duration trajectories were created by calculating the change between self‐reported sleep duration category [short (<7 hours), healthy (7‐9 hours) and long (>9 hours)] over a 4.5‐year period between enrollment and the first follow‐up visit (FU1) in SCCS. Survival time was calculated from 12 months after Medicare enrollment or FU1 to ADRD diagnosis, loss to follow‐up (Medicare coverage gaps>3 months), death or Dec.31^st^, 2020, whichever occurred first (Figure 1). Adjusted Cox proportional hazards regression models were used to estimate hazard ratios and 95% confidence intervals for incident ADRD.

**Result:**

Among 17,945 SCCS participants (median [IQR] age at FU1: 63 [59‐68] years), we identified 2,093 new ADRD cases during a median follow‐up of 5.1 years (IQR: 2.9‐8.2 years) in claims data. Adjusted cumulative incidence function curve displays the cumulative incidence of ADRD by sleep trajectory category (Figure 2). The long‐short trajectory had the highest cumulative ADRD incidence. Compared to the optimal sleep duration trajectory (maintaining 7‐9 hours in both reporting periods), suboptimal sleep duration trajectories were associated with a 20%–69% greater risk of ADRD: including the long‐short (HR=1.69, 95% CI=1.25‐2.27), long‐long (HR=1.56, 95% CI=1.21‐2.01), long‐healthy (HR=1.50, 95% CI=1.23‐1.82), short‐long (HR=1.49, 95% CI=1.16‐1.91), and short‐short (HR=1.20, 95% CI=1.06‐1.36) trajectories (Figure 3).

**Conclusion:**

Maintaining short and long sleep duration over time was associated with an increased risk of incident ADRD. Transitioning to shorter or longer sleep duration over time may also serve as a potential risk factor in the development of ADRD. Findings highlight the importance of maintaining healthy sleep duration throughout midlife to reduce incident ADRD risk.